# Human telomerase reverse transcriptase (hTERT) promotes gastric cancer invasion through cooperating with c-Myc to upregulate heparanase expression

**DOI:** 10.18632/oncotarget.6575

**Published:** 2015-12-11

**Authors:** Bo Tang, Rui Xie, Yong Qin, Yu-Feng Xiao, Xin Yong, Lei Zheng, Hui Dong, Shi-Ming Yang

**Affiliations:** ^1^ Department of Gastroenterology, Xinqiao Hospital, Third Military Medical University, Chongqing 400037, China; ^2^ Department of Medicine, School of Medicine, University of California, San Diego, CA 92093, USA; ^3^ Department of Nuclear Medicine, Southwest Hospital, Third Military Medical University, Chongqing 400038, China

**Keywords:** hTERT, heparanase, gastric cancer, invasion, c-Myc

## Abstract

Human telomerase reverse transcriptase (hTERT) is a central regulator of multiple hallmarks of tumors. However, the potential roles of hTERT in tumor invasion and metastasis and the underlying molecular mechanisms remain poorly understood. Here, we found that the expression of hTERT in gastric cancer (GC) was significantly associated with an advanced TNM stage, lymphatic metastasis. Survival analysis identified hTERT as an independent prognostic factor for survival of GC patients. hTERT promoted the invasion and metastasis of GC cells by binding to c-Myc and recruiting the complex to heparanase promoter to upregulate heparanase expression. In addition, our data demonstrated that hTERT activated Wnt/Î²-catenin signaling to promote c-Myc expression which could in turn activate hTERT transcription and expression, suggesting a positive feedback regulation in GC progression. Consistently, c-Myc and heparanase expression was positively correlated with hTERT levels, and was also an independent predictor of metastasis and survival. Collectively, our data provide a novel molecular mechanism for hTERT in promotion of GC invasion and metastasis, and highlight the molecular etiology and clinical significance of hTERT in GC progression. Targeting hTERT may represent a new therapeutic strategy to improve therapy and survival of GC patients.

## INTRODUCTION

Gastric cancer (GC) has become the fourth most common cancer and the second leading cause of cancer-related death worldwide [1, 2]. The prognosis of GC remains poor despite adequate surgery and chemotherapy because most GC patients present with advanced disease at the time of diagnosis [3]. Invasion and metastasis play critical roles in the progression of GC. However, the mechanisms of invasion and metastasis of GC remain poorly understood [4]. Thus, it is urgently needed for an improved understanding of the molecular mechanisms underlying GC invasion and metastasis.

Telomerase is a ribonucleoprotein complex that is composed of RNA and human telomerase reverse transcriptase (hTERT); it catalyzes the *de novo* synthesis of repetitive telomeric DNA after cell division and maintains chromosomal stability, leading to cellular immortalization [5]. The expression of hTERT was observed in 80%-90% of human tumors, and thus the targeting of telomerase or hTERT for cancer therapy has been suggested [6, 7]. In addition, there is an increasing evidence implicating hTERT in cancer, not only by maintaining telomeres but also *via* mechanisms independent of telomere lengthening [8, 9]. hTERT has been proposed to be a central regulator of multiple hallmarks of cancer, including proliferation, survival and self-renewal [10–12]. However, only limited research has been focused on the important role of hTERT in the invasion and metastasis of tumors [13, 14]. Therefore, it is of great importance to elucidate the regulatory mechanisms and biochemical properties of hTERT in cancer invasion and metastasis.

Heparanase (Hpa) is the only endogenous endoglycosidase that degrades heparan sulfate proteoglycans (HSPG) in the extracellular matrix (ECM) and basement membrane (BM), enabling tumor cells to break through these barriers for invasion and metastasis [15]. Cleavage of HSPG results in both physical remodeling of the ECM and the release of tethered growth factors such as bFGF, VEGF and HGF, which can strongly promote the angiogenesis, invasion and metastasis of tumors [16, 17]. Heparanase also possesses enzymatic-independent functions that can increase the transcriptional and protein levels of other genes associated with cell migration and invasion [18, 19]. However, few studies focused on the regulation of heparanase expression in cancer cells [20, 21].

In the present study, we aimed to investigate the mechanism by which hTERT enhanced the invasion and metastasis of GC cells. We demonstrate that hTERT can act as a co-activator to recruit the transcriptional factor c-Myc to the promoter region of heparanase to increase its expression, and finally enhances the invasion and metastasis of GC cells. Our findings provide novel insights into the crucial role of hTERT in GC progression and suggest hTERT as a potential target for the prevention of GC metastasis.

## RESULTS

### hTERT promotes GC invasion and metastasis and the expression of hTERT is positively correlated with heparanase in GC

Our previous studies have demonstrated that the expression of hTERT is associated with the proliferation and tumorigenesis of GC [22, 23]. We then compared the expression levels of hTERT and heparanase in GC cell lines, and found that hTERT and heparanase were expressed relatively higher in MKN45 cells while lower in SGC7901 cells (Supplementary Figure S1A). We selected these two cell lines in the following experiments. Consistent with the previous studies [24], overexpression of hTERT enhanced the invasion of GC cells, whereas knockdown of hTERT significantly inhibited the invasion *in vitro* (Supplementary Figure S1B and S1C). Stable GC cell lines were established and the protein expression was confirmed (Supplementary Figure S2A). Pulmonary metastasis and peritoneal dissemination assays were performed to test the metastasis of GC cells *in vivo*. Overexpression of hTERT increased the number of metastasis nodules in the lung and peritoneal cavity, while hTERT-knockdown repressed pulmonary metastasis and peritoneal dissemination *in vivo* (Supplementary Figure S2B and S2C).

**Figure 1:**
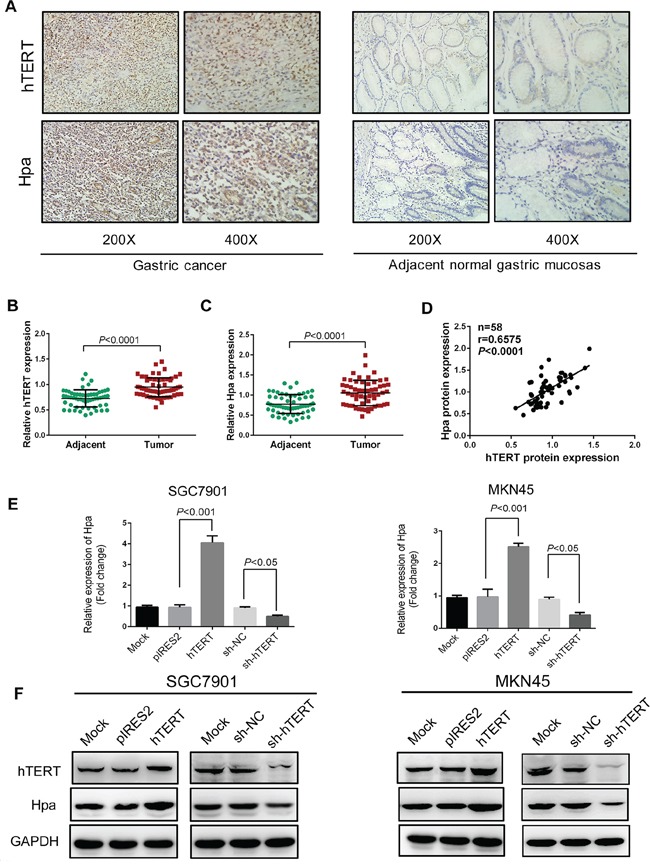
hTERT correlates significantly with heparanase in GC tissues and regulates heparanase expression in GC cells **A.** Immunohistochemical staining for the expression of hTERT and heparanase in gastric cancer and corresponding adjacent normal tissues. **B.** and **C.** Semi-quantitative analysis used to score the tissue sections, and relative expression levels of hTERT and heparanase determined in gastric cancer and corresponding adjacent normal tissues. **D.** Correlation of the protein expression levels of hTERT and heparanase were analyzed using Pearson's correlation analysis (*P* < 0.0001, r=0.6575). **E.** The mRNA levels of heparanase were determined by qPCR in SGC7901 and MKN45 cells transiently transfected with pIRES2, pIRES2-hTERT plasmids, or sh-RNA, or sh-hTERT vectors, respectively. **F.** The protein expression levels of hTERT and heparanase were examined by western blot analysis in SGC7901 and MKN45 cells transiently transfected with pIRES2, pIRES2-hTERT plasmids, or sh-RNA, or sh-hTERT vectors, respectively.

Since heparanase (Hpa) played a key role in invasion and metastasis of cancer cells [15–17], we then tested the expression levels of hTERT and Hpa by immunohistochemical assays (Figure 1A). The expression levels of hTERT and Hpa were clearly higher in GC tissues than those in adjacent normal tissues (Figure 1B and 1C). Furthermore, we found that hTERT expression was positively correlated with Hpa expression in GC tissues (Figure 1D). Taken together, hTERT could promote the invasion and metastasis of GC cells *in vitro* and *in vivo*, and the expression of hTERT was positively correlated with Hpa expression in GC tissues.

### hTERT upregulates heparanase expression in GC cells

Further, we investigated whether hTERT could regulate Hpa in GC cells. We quantitated Hpa expression in SGC7901 and MKN45 cells using qPCR and western blot analysis. Interestingly, we observed that the mRNA and protein levels of Hpa were markedly increased by overexpression of hTERT. Inversely, the expression of Hpa was reduced when hTERT was down-regulated by shRNA vectors (Figure 1E and 1F). Collectively, these findings suggested that hTERT exerted a positive role in regulating Hpa mRNA and protein expression in GC cells.

### hTERT upregulates the transcriptional activity of heparanase through the c-Myc binding site

**Figure 2:**
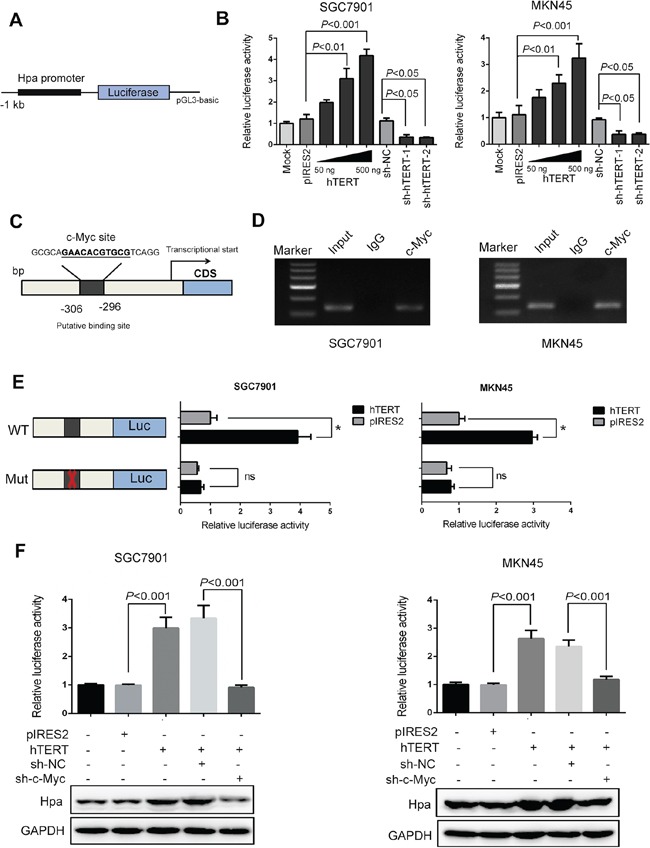
hTERT regulates the transcriptional activity of heparanase through the c-Myc binding site **A.** The 5′-flanking promoter region of the heparanase gene was cloned into the NheI/HindIII site of the luciferase construct pGL3-Basic vector. **B.** The relative luciferase activities of the heparanase promoter were tested using the dual luciferase assay in SGC7901 and MKN45 cells that were transiently transfected with pIRES2-hTERT (50, 200, 500 ng) and sh-hTERT-1 and sh-hTERT-2 vectors, respectively. **C.** The putative binding site of c-Myc on the heparanase promoter region was predicted using bioinformatics analysis. **D.** Chromatin immunoprecipitation (ChIP) assays were conducted in SGC7901 and MKN45 cells using an antibody against c-Myc or a control rabbit normal immunoglobulin G. An equivalent of amount of DNA in all of the samples served as an Input control. **E.** The luciferase activities of the heparanase promoter with wild type (or the mutant binding site of c-Myc) were determined using luciferase reporter assays in SGC7901 and MKN45 cells that were transiently transfected with pIRES2, pIRES2-hTERT plasmids. (*P < 0.001, ns denotes no significance). **F.** Luciferase reporter assays and western blot analysis were performed to examine the promoter activities and protein expression levels of heparanase in SGC7901 and MKN45 cells.

We evaluated the effect of hTERT on Hpa promoter activity using a luciferase reporter driven by the Hpa promoter (Figure 2A). As shown in Figure 2B, the transcriptional activity of Hpa gene promoter was significantly increased by hTERT in a dose-dependent manner. In contrast, the promoter activity of Hpa was attenuated when hTERT expression was inhibited by shRNA-1 or shRNA-2 (Figure 2B), suggesting that hTERT increases Hpa expression by upregulating its promoter activity. Next, we examined the mechanism by which hTERT activated the Hpa promoter. We know that hTERT indirectly exerts its transcriptional regulation by binding to other nuclear transcriptional factors [25–26]. Therefore, we used three promoter analysis programs (TF Search, Alggen and JASPAR) to explore the putative transcription factors that could bind to the Hpa promoter. Six putative transcription factors were identified using the analysis programs with high scores (Supplementary Figure S3A). Next, the siRNAs of these six factors were synthesized, and the inhibitory effect was confirmed using qPCR (Supplementary Figure S3B). The promoter activity of Hpa was reduced to the greatest extent when c-Myc expression was inhibited (Supplementary Figure S3C). Additionally, Hpa mRNA expression was markedly decreased when the siRNA of c-Myc was transfected in cells (Supplementary Figure S3D). Thus, we speculated that c-Myc might play an important role in the transcriptional regulation of Hpa by hTERT.

To further determine whether c-Myc is associated with Hpa promoter activity in GC cells, we constructed c-Myc overexpression plasmids and shRNA vectors. Cells were transfected with these plasmids or vectors, and then the transfection efficiency was examined by western blot analysis (Supplementary Figure S4A and S4B). As shown in Supplementary Figure S4C, Hpa promoter activity was significantly increased by overexpression of c-Myc in a dose-dependent manner. Conversely, the promoter activity was significantly decreased by knockdown of c-Myc (Supplementary Figure S4C). Similarly, the protein expression was increased or reduced by the up- or down-regulation of c-Myc (Supplementary Figure S4E and S4F). Next, the putative binding site of c-Myc in the promoter region of Hpa was predicted using bioinformatics (Figure 2C). To confirm the specificity of c-Myc in the activation of Hpa promoter, we cloned the Hpa promoter without the putative c-Myc-binding sequence. The mutation resulted in a significant reduction of promoter activity (Supplementary Figure S4D). The chromatin immunoprecipitation (ChIP) assay showed that c-Myc could bind to the promoter of Hpa, which confirmed that c-Myc was associated with Hpa promoter region *in vivo* (Figure 2D). Moreover, the promoter activity of Hpa was greatly decreased when the c-Myc binding site was mutated or c-Myc expression was suppressed (Figure 2E and 2F). Additionally, the increased protein expression level was also attenuated by inhibiting c-Myc expression (Figure 2F). These results indicated that hTERT regulation of the Hpa promoter activity was c-Myc-dependent.

### hTERT interacts with c-Myc and recruits c-Myc to transactivate Hpa expression

**Figure 3:**
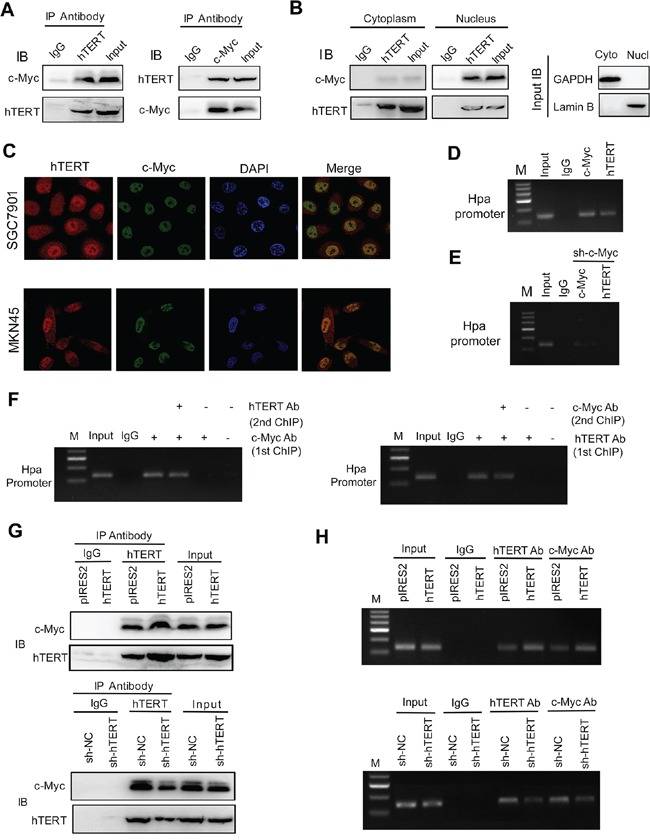
hTERT interacts with c-Myc and regulates the binding of the hTERT/c-Myc complex to the heparanase promoter **A.** The total cell lysates of SGC7901 cells were prepared for immunoprecipitation (IP) with antibodies against hTERT or c-Myc, respectively. Rabbit IgG served as a negative control. An aliquot of cell lysate was used as an Input control. **B.** Cytoplasm and nuclear lysates of SGC7901 cells were prepared for IP with anti-hTERT or anti-c-Myc and then evaluated by IB. **C.** The subcellular localization and the colocalization of hTERT and c-Myc were tested by dual immunofluorescence using confocal microscopy. **D.** hTERT and c-Myc co-existed in the heparanase promoter region. ChIP assays were performed using antibody against c-Myc or hTERT. **E.** Binding of the c-Myc/hTERT complex to promoter was mediated by c-Myc. SGC7901 cells were transfected with sh-c-Myc vectors, and then ChIP was performed using an antibody against c-Myc or hTERT. **F.** hTERT and c-Myc were present in the same protein complex on the heparanase promoter. Soluble chromatin from SGC7901 cells was first incubated with anti-c-Myc or anti-hTERT antibodies. The ReChIP assay was similarly processed using anti-hTERT or anti-c-Myc, respectively. **G.** hTERT regulates the interaction of c-Myc and hTERT. Co-IP was performed and evaluated by IB with the indicated antibodies. **H.** hTERT regulates the recruitment of c-Myc to the heparanase promoter. SGC7901 cells were transfected with vectors as indicated. Soluble chromatin was prepared for the ChIP assays.

The above results demonstrated that hTERT could exert its transcriptional regulation of Hpa through c-Myc binding activity. Thus, it is logical to postulate that hTERT cooperates with c-Myc to occupy the promoter region of Hpa. We showed that hTERT and c-Myc could bind to one another (Figure 3A, Supplementary Figure S5A). Immunoprecipitation demonstrated that colocalization of hTERT and c-Myc mainly occurred in the nucleus (Figure 3B, Supplementary Figure S5B). The results of dual immunofluorescence also showed that hTERT colocalized with c-Myc in the nucleus (Figure 3C). As shown in Figure 3D, a band of the predicted size was detected in the c-Myc binding region using anti-hTERT or anti-c-Myc antibodies, indicating a binding role of hTERT and c-Myc in the Hpa promoter. The bindings of both hTERT and c-Myc to the Hpa promoter were significantly attenuated by knockdown of c-Myc (Figure 3E), suggesting that the binding was mediated by c-Myc. To confirm whether the hTERT/c-Myc complex was directly involved in binding to the Hpa promoter, reChIP assays were performed. The data in Figure 3F demonstrated that both hTERT and c-Myc simultaneously bound to the c-Myc binding site in the Hpa promoter.

Up-regulation of hTERT could promote the interaction of hTERT with c-Myc (Figure 3G). In contrast, knockdown of hTERT markedly suppressed the interaction of hTERT with c-Myc in SGC7901 cells (Figure 3G), suggesting that hTERT is required for the physical interaction between hTERT and c-Myc. The same results were obtained in MKN45 cells (Supplementary Figure S5C and S5D). Interestingly, the interaction of hTERT and c-Myc was increased considerably in GC cells compared with normal gastric cells (Supplementary Figure S6A). Moreover, we found that the GC cell lines had strong binding activity of hTERT and c-Myc on heparanase promoter compared with normal gastric cells (Supplementary Figure S6B). Dual immunofluorescence showed that hTERT colocalized with c-Myc and the intensity in GC tissues was much higher than that in the adjacent normal tissues (Supplementary Figure S6C). As shown in Figure 3H, hTERT overexpression increased the binding of hTERT and c-Myc to the Hpa promoter; while depletion of hTERT resulted in reduced binding to the promoter region, indicating that hTERT could promote the formation of the transcriptional complex and recruit c-Myc to the Hpa promoter and, consequently, increase the expression of Hpa in GC cells.

### The intact complex of hTERT and c-Myc is essential for Hpa expression

**Figure 4:**
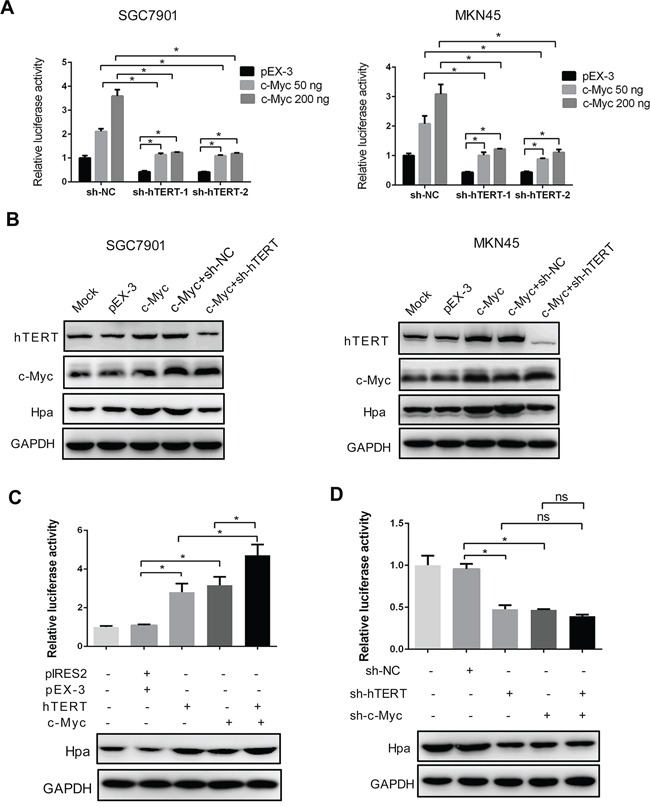
The intact hTERT and c-Myc complex is essential for heparanase promoter activity and expression **A.** and **B.** hTERT knockdown impaired c-Myc-enhanced heparanase promoter activities and protein expression. SGC7901 and MKN45 cells were transiently transfected with pEX-3 or c-Myc plasmids, and sh-hTERT-1 or sh-hTERT-2 as indicated, respectively. The relative luciferase activities of the heparanase promoter and protein expression were examined using the dual luciferase assay and western blot analysis. (*P < 0.001). **C.** hTERT and c-Myc had a synergetic effect in promoting the expression of heparanase. The luciferase reporter assay and western blot analysis were performed as described (*P < 0.001). **D.** Knockdown of hTERT, c-Myc or both reduced the promoter activity and protein expression of heparanase. The luciferase reporter assay and western blot analysis were performed as described. *P < 0.001, ns denotes no significance.

We investigated whether the promoter activity of Hpa was dependent on the intact complex of hTERT and c-Myc. Interestingly, we found that the elevated promoter activity induced by overexpression of hTERT or c-Myc could be abolished by the c-Myc shRNA or hTERT shRNA in SGC7901 and MKN45 cells (Figure 2F and Figure 4A). Similarly, knockdown of hTERT inhibited the c-Myc-stimulated expression of Hpa (Figure 4B). These results strongly suggested that the promoter activity of Hpa was closely dependent on the interaction between these two proteins. Furthermore, we transiently transfected hTERT, c-Myc plasmids or the combination of these two plasmids into cells and performed luciferase assays. Consistently, either hTERT or c-Myc overexpression could increase the promoter activity of Hpa. Moreover, overexpression of both hTERT and c-Myc could stimulate much higher promoter activity and protein expression (Figure 4C). Conversely, either hTERT shRNA or c-Myc shRNA reduced the promoter activity and protein expression, whereas the combination of these two shRNAs was not able to further reduce Hpa expression (Figure 4D). Taken together, these findings demonstrated that hTERT might act as a co-activator of c-Myc to transactivate the promoter activity of Hpa, and the intact complex is essential for Hpa expression.

### hTERT regulates c-Myc expression through the Wnt/β-catenin pathway

**Figure 5:**
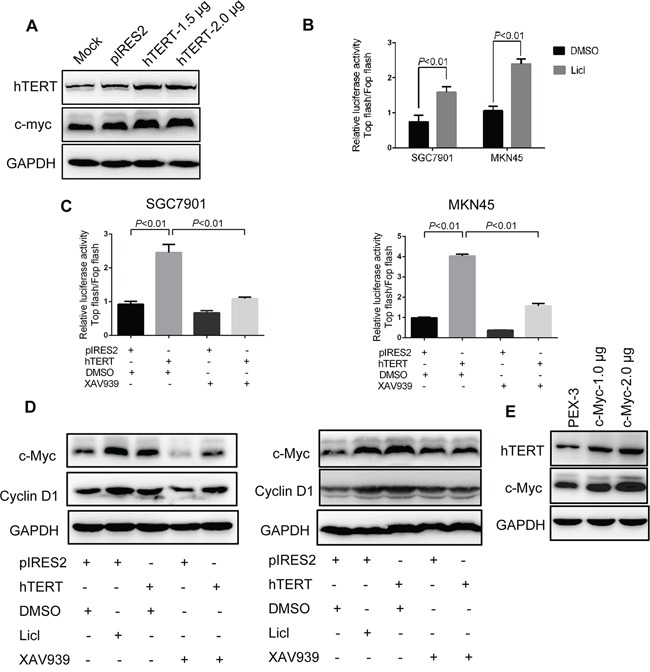
hTERT up-regulates c-Myc expression by activating Wnt/β-catenin signaling **A.** Overexpression of hTERT could up-regulate the protein expression of c-Myc. Western blot analysis was performed to detect the expression of c-Myc and hTERT as indicated. **B.** Wnt/β-catenin signaling could be activated in SGC7901 and MKN45 cells. Cells were treated with LiCl, and TCF/LEF reporter activity was assessed using the dual reporter assay kit. **C.** hTERT promoted TCF/LEF reporter activity. SGC7901 and MKN45 cells were transiently transfected with pIRES2, pIRES2-hTERT plasmids and then treated with XAV939. **D.** hTERT promoted the expression of the target gene of Wnt/β-catenin. The protein expression levels of c-Myc and Cyclin D1 were examined by western blot analysis. GAPDH was used as an internal control. **E.** c-Myc could upregulate the expression of hTERT. Western blot analysis was performed to detect the expression of hTERT and c-Myc.

Interestingly, we found that c-Myc protein expression was increased by overexpression of hTERT (Figure 5A). c-Myc is an important downstream target gene of the Wnt/β-catenin pathway [27–28]. Thus, we investigated whether hTERT could activate the Wnt/β-catenin pathway to further increase the expression level of c-Myc. First, we observed that Wnt/β-catenin pathway could be activated in SGC7901 and MKN45 cells (Figure 5B). Next, hTERT overexpression markedly increased the activity of β-catenin, while the elevated activity could be abolished by Wnt inhibitor XAV939 (Figure 5C). The immunofluorescence illustrated marked translocation of β-catenin into the nucleus in response to hTERT overexpression (Supplementary Figure S7A). Moreover, the expression of β-catenin decreased in the cytoplasm but increased in the nucleus, further suggesting that overexpression of hTERT increased the translocation of β-catenin (Supplementary Figure S7B). Next, we evaluated the downstream targets of the Wnt/β-catenin pathway using western blot analysis. As shown in Figure 5D, overexpression of hTERT significantly increased the expression of Cyclin D1 and c-Myc. Many studies demonstrated that c-Myc could bind to the hTERT promoter and transcriptionally regulate hTERT expression (29). In line with previous studies, overexpression of c-Myc could increase hTERT expression (Figure 5E). Thus, we concluded that a feedback regulation mechanism occurred between hTERT and c-Myc in GC cells, which might represent an important point of regulation in the development of GC.

### hTERT promotes the invasion and metastasis of GC cells *via* c-Myc and heparanase *in vitro* and *in vivo*

**Figure 6:**
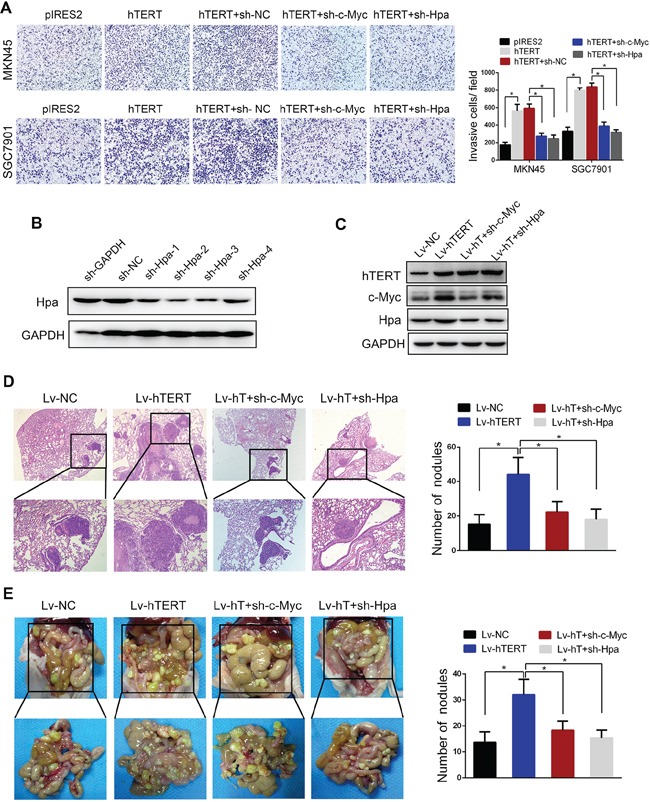
hTERT promotes the invasion and metastasis of gastric cancer cells through c-Myc and heparanase *in vitro* and *in vivo* **A.** c-Myc and heparanase knockdown impaired hTERT-enhanced invasion of gastric cancer cells *in vitro*. The invasive properties of the cells were analyzed using an invasion assay with a Matrigel-coated plate. All of the experiments were performed at least three times (*P* < 0.001). **B.** and **C.** Western blot analysis was performed to confirm the transfection efficiency of sh-Hpa and the lentivirus vectors. **D.** c-Myc and heparanase knockdown abolished hTERT-enhanced tumor pulmonary metastasis. The black circles in the sections reveal the tumor focus that was formed in the lung. The histogram shows the calculated number of metastasis nodules (*P* < 0.01). **E.** c-Myc and heparanase knockdown abolished hTERT-enhanced tumor peritoneal dissemination. SGC7901 cells transfected with the indicated vectors were injected into nude mice. The number of tumor nodules was calculated (*P* < 0.01).

The invasion assay demonstrated that c-Myc and Hpa knockdown significantly abolished hTERT-enhanced invasion (Figure 6A and 6B). To further evaluate the effect of c-Myc and Hpa in hTERT-enhanced metastasis of GC cells *in vivo*, pulmonary metastasis and peritoneal dissemination assays were performed. SGC7901-hTERT cells were transfected with c-Myc and Hpa shRNA vectors, and the protein expression levels were confirmed (Figure 6C). The data showed that c-Myc and Hpa knockdown markedly attenuated hTERT-induced metastasis in lung and peritoneal cavity (Figure 6D and 6E). Meanwhile, the expression levels of hTERT and Hpa in tumor nodules from pulmonary metastasis and peritoneal dissemination were analyzed by western blot and immunohistochemistry analysis (Supplementary Figure S8). The data showed that the expression levels of hTERT were positively associated with Hpa expression, consistent with our previous data obtained in human GC tissues. Thus, we concluded that hTERT significantly promoted the invasion and metastasis of GC cells *via* c-Myc and heparanase both *in vitro* and *in vivo*.

### hTERT, c-Myc and Hpa expression levels are positively related and associated with poor prognosis in GC

**Figure 7:**
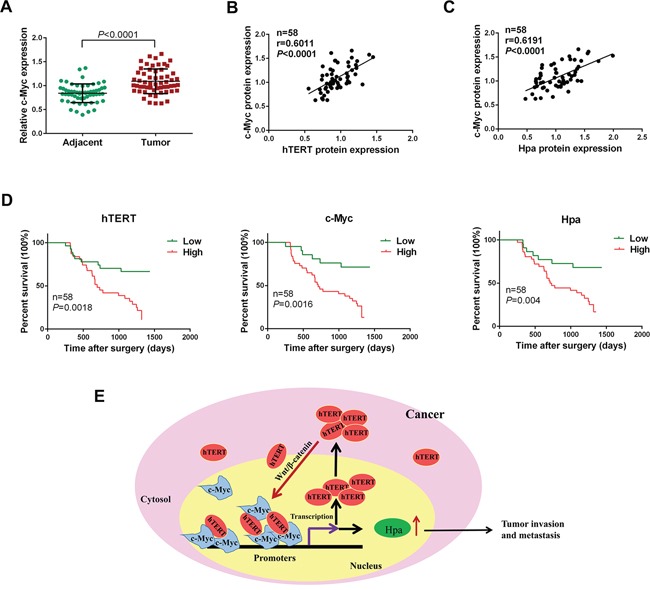
hTERT, c-Myc and Hpa expression levels are positively related and associated with a poor prognosis in gastric cancer **A.** The expression level of c-Myc was semi-quantitatively analyzed using immunochemistry. The relative expression level of c-Myc was determined in gastric cancer and corresponding adjacent normal tissues (*P* < 0.0001). **B.** and **C.** Correlation of the protein expression levels of hTERT, c-Myc and heparanase were analyzed using Pearson's correlation analysis. **D.** Kaplan-Meier analysis of overall survival in GC patients with different hTERT, c-Myc and heparanase expression. **E.** Model for the transcriptional regulation of heparanase by hTERT in GC cells. hTERT translocates to the nucleus and binds to c-Myc to form a transcriptional complex, which then binds to heparanase promoter to promote its expression. hTERT upregulates c-Myc expression by activating Wnt/β-catenin signaling. In turn, c-Myc binds directly to hTERT promoter to transactivate hTERT, which forms a positive feedback loop to sustain high levels of hTERT as well as c-Myc.

We showed that c-Myc was expressed at a higher level in GC tissues compared with adjacent normal tissues (Figure 7A). The expression of c-Myc was positively correlated with both hTERT and Hpa in GC (Figure 7B and 7C). As shown in Table 1, high expression levels of hTERT and Hpa were closely correlated with an advanced TNM stage and lymphatic metastasis. The survival analysis revealed that high expression levels of hTERT, c-Myc and Hpa were strongly associated with a decreased survival time and poor prognosis (Figure 7D), suggesting an important role of hTERT, c-Myc and Hpa expression in predicting the prognosis of patients with gastric cancer.

**Table 1:** Relationship between hTERT, Hpa expression and clinicopathological features of GC patients

Clinicopathlogical factors	n	hTERT		*P*	Hpa		*P*
		Low	High		Low	High	
**Gender**							
Female	21	6	15	0.552	8	13	0.985
Male	37	8	29		14	23	
**Age (years)**							
≥55	30	7	23	0.882	10	20	0.455
<55	28	7	21		12	16	
**Tumor size**							
≥3cm	42	6	36	0.005*	14	28	0.369
<3cm	16	8	8		8	8	
**Differentiation**							
Moderate	18	3	15	0.372	4	14	0.098
Poorly	40	11	29		18	22	
**Lymph node metastasis**							
Positive	39	6	33	0.026*	10	29	0.006*
Negative	19	8	11		12	7	
**TNM stage**							
I - II	20	8	12	0.041*	12	8	0.01*
III - IV	38	6	32		10	28	

* *P* < 0.05

## DISCUSSION

Although invasion and metastasis are the most common cause of death in GC patients, the underlying mechanisms remain poorly understood. It has long been well established that hTERT expression and telomerase activation contribute to cellular immortalization, proliferation, and malignant transformation [11, 12]. To date, only a few studies have focused on the critical role of hTERT in cancer invasion and metastasis [30]. In the present study, our data provide strong evidence supporting the notion that hTERT plays a significant role in promoting invasion and metastasis of GC cells. Heparanase is a well-known pro-metastatic molecule with a high activity in most tumor cells. Its function is mainly dependent on the activity of heparanase and the regulation of other metastasis-related factors [31, 32]. We found that the expression of hTERT and heparanase were up-regulated in GC tissues, and the expression levels of hTERT are positively correlated with heparanase expression. Ectopic expression and knockdown of hTERT could markedly increase and reduce heparanase expression in GC cells, suggesting that hTERT was closely associated with the transcriptional regulation of heparanase in GC cells.

To our knowledge, hTERT is not a common nuclear transcriptional factor, which regulates other target genes by interacting with some other transcriptional factors. Previous studies demonstrated that hTERT was able to interact with BRG1 to activate Wnt-dependent target genes [25], bind to the NF-κB p65 subunit [26], and interact with β-catenin to enhance its nuclear localization and transcriptional activity [13]. Based on these studies, we investigated whether hTERT could function with other factors to promote the transcriptional expression of heparanase. We searched for the putative transcription factors that might bind to heparanase promoter region using three different bioinformatics programs. Several factors were obtained and subjected to further analysis. Intriguingly, we found that the hTERT-enhanced promoter activity and heparanase expression were significantly abolished by knockdown of c-Myc, indicating that c-Myc might be involved in the transcriptional regulation of heparanase. Furthermore, our data showed that c-Myc overexpression and depletion induced and decreased the promoter activity and heparanase expression, respectively. ChIP analysis revealed that c-Myc bound to the promoter of heparanase in GC cells. Interestingly, the hTERT or c-Myc-enhanced promoter activities were markedly abolished when the binding site of c-Myc was mutated, further demonstrating that hTERT-induced heparanase promoter activity was c-Myc-dependent.

The c-Myc proto-oncogene belongs to the MYC family of genes and is commonly activated in tumorigenesis [33–34]. c-Myc is overexpressed and/or activated in more than half of human cancers, and it appears to be a molecular hallmark of carcinogenesis [35]. c-Myc is a relatively weak transcriptional activator that can activate transcription by cooperating with many other positive factors to initiate tumorigenesis [36]. Many oncogenes were first discovered as cooperating events in screens of c-Myc-induced tumor development, and c-Myc has recently been suggested to be a global regulator of oncogenes [37]. In addition, the involvement of c-Myc in such a broad spectrum of cellular activities can be explained by the finding that c-Myc binds to 10-15% of all promoter regions [38]. Consistent with these theories, our data revealed that c-Myc could bind to the promoter region of heparanase. Thus, we hypothesized that hTERT might act as a positive regulator that interacts with c-Myc to initiate heparanase transcription. Up-regulation of hTERT could promote the interaction of hTERT with c-Myc, while knockdown of hTERT markedly inhibited formation of the complex. Moreover, hTERT could promote the occupancy of hTERT and c-Myc on the heparanase promoter, suggesting that hTERT promoted the formation of the transcriptional complex and recruit additional c-Myc to the heparanase promoter region. More importantly, we found that both hTERT and c-Myc were essential for the transcriptional expression of heparanase, and the promoter activity of heparanase depended closely on the interaction of these two proteins. These findings suggest that hTERT might act as a co-activator of c-Myc to transactivate the promoter activity of heparanase and that the intact complex of hTERT and c-Myc is essential for heparanase expression in GC cells.

Feedback regulation is an important mechanism of regulation in cancer [39, 40]. Interestingly, we found that c-Myc expression was increased by overexpression of hTERT. Thus, we speculated that hTERT was capable of upregulating c-Myc expression. Our data showed that hTERT activated the Wnt/β-catenin signaling to promote c-Myc expression. Importantly, previous studies showed that c-Myc could be recruited to hTERT promoter and subsequently activate hTERT gene expression [29, 41, 42]. Thus, the collective data suggest the presence of a feedback regulation between hTERT and c-Myc, which might be an important regulation in the progression of GC. Furthermore, our results demonstrated that knocdown of c-Myc and heparanase was able to attenuate hTERT-enhanced invasion and metastasis of GC cells. In addition, the expression levels of hTERT, c-Myc and heparanase in GC tissues were positively correlated with each other, and high expression levels of hTERT and heparanase were closely related to the advanced TNM stage and lymphatic metastasis. Moreover, the high expression levels of hTERT, c-Myc and heparanase were strongly associated with poor survival in GC patients, suggesting an important role of hTERT, c-Myc and heparanase expression in predicting the prognosis of GC patients.

Interestingly, our previous study found that hTERT could enhance FOXO3a ubiquitination and alleviate the inhibitory effect of FOXO3a on ITGB1 expression, which finally upregulated ITGB1 expression and tumor invasion [24]. We believe that this is not the only way that hTERT exerts its important role in tumor invasion and metastasis. Here, we found another important mechanism in which hTERT promotes the invasion and metastasis of GC cells by binding to c-Myc and recruiting the complex to heparanase promoter to upregulate heparanase expression (Figure 7E). It is well-known that tumor metastasis is a complicated process mainly including reduced adhesion, degradation of extracellular matrix, EMT and angiogenesis. In our previous study, we mainly focused on the ITGB1 expression which is a main component of cell adhesion molecules. To fully understand the role of hTERT in tumor metastasis, we sought to focus on the effect of hTERT on heparanase expression that is an important endogenous endoglycosidase degrading extracellular matrix in the present study. In view of the extensive regulation of hTERT in tumor, we think it is extremely important to fully elucidate the molecular mechanism of hTERT in tumor invasion and metastasis.

In conclusion, our data highlight the molecular etiology and clinical significance of hTERT in GC, and the expression levels of hTERT, c-Myc and heparanase in GC tissues were positively correlated and strongly associated with poor survival in GC patients. Thus, targeting hTERT may represent a new therapeutic strategy to improve therapy and survival for GC patients.

## MATERIALS AND METHODS

### Ethics statement

The study was approved by the Clinical Research Ethics Committee of the Third Military Medical University. Informed consent was obtained from each patient. All of the animal experimental procedures were performed according to the “Guide for the Care and Use of Laboratory Animals” published by National Institutes of Health and Ethics Committee of the Third Military Medical University.

### Plasmids, lentiviral constructs, and shRNA expressing vectors

The hTERT overexpression plasmid pIRES2-hTERT or control pIRES2-Neo plasmid were designed and synthesized by Cyagen (Cyagen Biosciences Inc., Guangzhou, China). The pEX-3-c-Myc expression plasmid was constructed by cloning the entire c-Myc coding fragment into the BglII/EcoRI site of the pEX-3 vector. The pEX-3-c-Myc overexpression plasmid and control plasmid were synthesized by GenePharma (Shanghai, China). Lentiviral constructs containing hTERT expression vector were obtained from GeneChem (Shanghai, China). The packaged lentiviruses were referred as Lv-hTERT and Lv-NC. The hTERT, c-Myc and heparanase short hairpin RNAs and control RNAs were designed and synthesized by GenePharma (Shanghai, China). The sequences of these shRNAs were listed in Supplementary Table S1.

### Cell culture and transfection

MKN45, SGC7901, and GES cells were obtained from the Chinese Academy of Sciences (Shanghai, China). Cells were maintained in Dulbecco's Modified Eagle's Medium (DMEM) (Hyclone, Waltham, MA, USA) supplemented with 10% fetal bovine serum at 37°C in a humidified atmosphere containing 5% CO_2_. For stable transfection, the titer of lentivirus was determined with serial dilution method. Cells were seeded into 96-well plates, followed by addition of 1 × 10^8^ TU/ml lentivirus (10 μl), 5 μg/ml polybrene (Sigma, St. Louis, MO, USA) and complete medium. Cells were subsequently selected for antibiotic resistance, and observed under a fluorescence microscope to evaluate the transfection efficiency. For transient transfection, the overexpression plasmids or shRNA vectors were transfected with lipofectamine 2000 (Invitrogen, Carlsbad, CA, USA) following the manufacturer's instructions. Then, cells were collected 48h after transfection for western blot analysis and qPCR, or treated for cell invasion assays.

### Immunohistochemistry and analysis

The tissues were embedded and blocked with 2.5% hydrogen peroxide in methanol and then incubated with rabbit anti-hTERT (Abcam, Cambridge, MA, USA), anti-heparanase (Santa Cruz, CA, USA), or anti-c-Myc (Abcam, Cambridge, MA, USA) antibodies at 4°C overnight. After incubation with the secondary antibody, immunostaining was performed using diaminobenzidine. The staining results were observed by microscopy, and ImagePro Plus (Media Cybernetics, MD, USA) was used to quantitatively score the tissue sections. The detailed procedure was performed as described previously in our laboratory [43].

### Real-time RT-PCR

Quantitative PCR was conducted using SYBR Premix EX Taq (Takara, Dalian, China) and an ABI detection system (Applied Biosystems, Foster City, CA, USA) with GAPDH expression as the internal control. All of the reported results are the average ratios of three independent experiments.

### Western blot analysis

Whole-cell and tissue extracts were prepared, and the protein concentration was measured using a Bradford Protein Assay Kit (Beyotime, Beijing, China). The transferred membranes were subsequently incubated with primary rabbit anti-hTERT, anti-c-Myc, anti-heparanase, anti-Cyclin D1 (Santa Cruz, CA, USA) and mouse monoclonal anti-GAPDH (Abcam, Cambridge, MA, USA) antibodies. The protein bands were detected using chemiluminescence (Millipore, Temecula, CA, USA).

### Construction of luciferase vectors

The 5′-flanking promoter region of the heparanase gene was cloned into the NheI/HindIII site of the luciferase construct pGL3-Basic vector (Hpa-wt-Luc). The Hpa-mut-Luc without the c-Myc element was generated by one-step opposite direction PCR with the Mutant Best Kit (Takara, Dalian, China). The expected mutations and integrity of the vector were confirmed by direct sequencing (Sangon, Shanghai, China).

### Luciferase reporter assay

The cells were seeded in 24-well plates and transfected with 500 ng Hpa-wt-Luc or Hpa-mut-Luc reporter vectors using Lipofectamine (Invitrogen, Carlsbad, CA, USA). For the Wnt reporter assay, cells were seeded into 24-well plates and transfected with 200 ng TOPflash or FOPflash expression plasmids (Millipore, Temecula, CA, USA) or co-transfected with hTERT, c-Myc, sh-RNA palsmids as indicated using lipofectamine 2000. To examine Wnt activity, cells were treated with Wnt activator Licl (10 mM) (R&D systems, Abingdon, UK) or Wnt inhibitor XAV939 (10 μM) (Sigma-Aldrich, St. Louis, MO, USA). The cell extracts were harvested after 36 h and lysed with passive lysis buffer. The luciferase activity was then measured using the Dual Luciferase Reporter assay (Promega, Madison, WI, USA).

### Chromatin Immunoprecipitation (ChIP) and reChIP assay

ChIP assays were performed using a ChIP assay kit (Active Motif, Carlsbad, CA, USA) according to the manufacturer's instructions. An equivalent amount of DNA in all of the samples was used as the Input control. Subsequently, the protein-DNA complex was immunoprecipitated with anti-hTERT, anti-c-Myc and mouse IgG as a negative control antibody. The chromatin reimmunoprecipitation (reChIP) assays were performed according to the protocol provided with the manual (Active Motif, Carlsbad, CA, USA). The PCR products were resolved in a 2% agarose gel and visualized by ethidium bromide staining. All of the experiments were performed in triplicate.

### Co-immunoprecipitation assay

Whole cell extraction and nuclear extraction were performed according to the instruction manual (Active Motif, Carlsbad, CA, USA). A quantity of 500 μg nuclear or whole extract was combined with 5 μg anti-hTERT or anti-c-Myc antibody and incubated for 4 h at 4°C. Subsequently, 25 μl Protein G Magnetic Beads (Active Motif, Carlsbad, CA, USA) was added. The Protein G precipitated protein complex was carefully recovered using a magnetic stand followed by four washes with Complete Co-IP/Wash Buffer (Active Motif, Carlsbad, CA, USA). The harvested samples were analyzed by western blot analysis.

### Immunofluorescence

SGC7901 and MKN45 cells were grown on coverslips. The cells were washed with PBS and fixed with 4% paraformaldehyde (w/v) for 30 min, and then permeabilized with 0.5% Triton X-100 in PBS and blocked with BSA. The cells were incubated with primary rabbit anti-β-catenin (Abcam, Cambridge, MA, USA), anti-hTERT and mouse anti-c-Myc antibodies at 4°C overnight. After careful washing with PBS, the cells were incubated with secondary antibodies conjugated to Cy3 or FITC. Fluorescent images were observed and analyzed with a laser scanning confocal microscope (Olympus, Japan).

### Cell invasion assay

The bottom of the transwell chamber was coated with BD Matrigel Basement Membrane Matrix (BD Biosciences, San Diego, CA, USA). The cells were harvested and placed in the upper chamber containing 5% fetal bovine serum. The lower chamber was filled with DMEM containing 25% fetal bovine serum. Invading cells were fixed with 4% paraformaldehyde, stained with crystal violet and dried. All of the experiments were repeated three times.

### *In vivo* metastasis assay

SGC7901 cells were transfected with the lentiviral vectors Lv-NC or Lv-hTERT, and the SGC7901-Lv-NC and SGC7901-Lv-hTERT cells were further transfected with sh-hTERT or sh-Hpa plasmids, respectively. The transfection efficiency was confirmed by western blot analysis. For the *in vivo* lung metastasis assay, 1 × 10^6^ transfected cells were suspended in 200 μl phosphate-buffered saline for each mouse. The cells were injected into nude mice (five mice per group, five-week-old male BALB/c-nu/nu mice) through the tail vein. Each lung was divided into five blocks, and the paraffin mass was cut into five sections and stained with hematoxylin and eosin. The metastatic nodules were observed under a microscope and calculated. For the peritoneal dissemination assay, SGC7901 cells were transfected as indicated above, and 1 × 10^6^ transfected cells were injected into the abdominal cavity of nude mice (five mice per group, five-week-old male BALB/c-nu/nu mice). Six weeks later, the mice were killed, and the nodules were observed and counted.

### Statistical analysis

Data from the statistical analysis represent the mean ± standard deviation (SD) from at least three independent experiments. A two-tailed Student's *t*-test or Mann-Whitney test was used to determine differences between groups. The correlation coefficients were determined using Pearson's rank correlation test. Kaplan-Meier methodology was used for the survival analysis. *P* < 0.05 was considered statistically significant. All of the statistical analyses were performed using SPSS 17.0 software, and the graphs were generated using GraphPad Prism 6.0 (Graphpad Software Inc, CA, USA).

## SUPPLEMENTARY FIGURES AND TABLE


