# Supplementary material for: Human telomerase reverse transcriptase (hTERT) promotes gastric cancer invasion through cooperating with c-Myc to upregulate heparanase expression
Source: Oncotarget. 2015 Dec 11;7(10):11364–79. doi: 10.18632/oncotarget.6575 (PMC4905479; doi:10.18632/oncotarget.6575)
Supplement: Supplementary file 1 [file oncotarget-07-11364-s001.pdf]

# SUPPLEMENTARY FIGURES AND TABLE

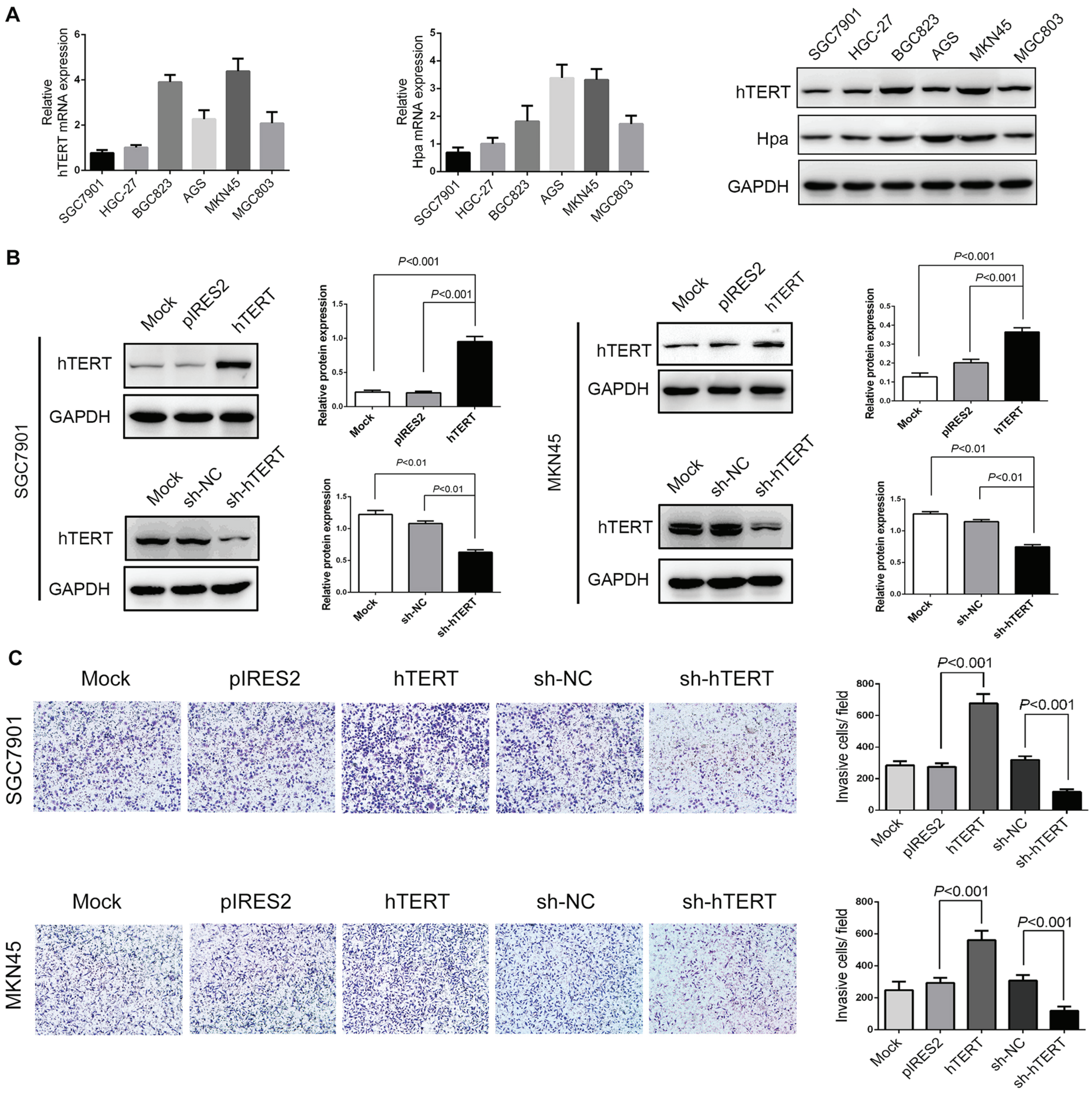


**Supplementary Figure S1: hTERT promoted invasion of gastric cancer cells *in vitro*. A.** The expression levels of hTERT and heparanase in gastric cell lines were examined using qPCR and western blot analysis. **B.** The transfection efficiency was assessed using western blot analysis. SGC7901 and MKN45 cells were transfected with pIRES2, pIRES2-hTERT plasmids, or sh-RNA, or sh-hTERT vectors, respectively. Next, the protein expression levels of hTERT were detected by western blot analysis. **C.** hTERT promoted the invasion of gastric cancer cells *in vitro*. SGC7901 and MKN45 cells were transfected with pIRES2, pIRES2-hTERT plasmids, sh-RNA, or sh-hTERT vectors, respectively. The invasive properties of the cells were analyzed using an invasion assay with a Matrigel-coated plate. All of the experiments were performed at least three times ($P < 0.001$).

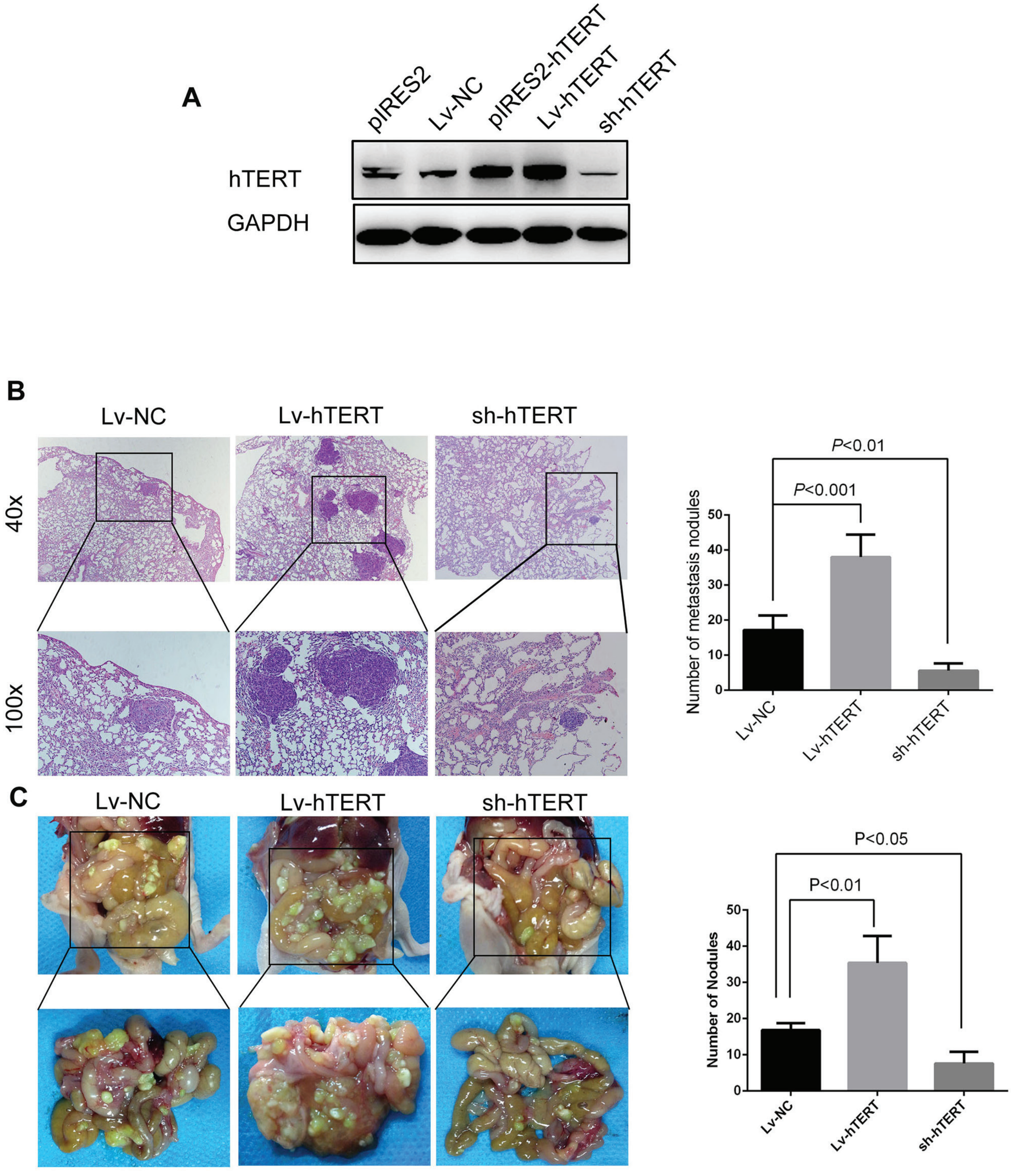


**Supplementary Figure S2: hTERT promoted the metastasis of gastric cancer cells *in vivo*. A.** SGC7901 cells were transfected with lentivirus-NC, lentivirus-hTERT or sh-hTERT vectors, respectively. The protein expression levels were evaluated using western blot analysis. **B.** hTERT enhanced the pulmonary metastasis of gastric cancer cells. SGC7901 cells transfected with the indicated vectors were injected into the tail vein of nude mice. The mice were killed, and the lungs were isolated for hematoxylin and eosin staining. The black circles in the sections indicate the tumor foci formed in the lung. The histogram shows the calculated number of metastasis nodules. Each group contains five nude mice. **C.** hTERT promoted tumor peritoneal dissemination. SGC7901 cells transfected with the indicated vectors were injected into the nude mice. The tumor nodules were calculated.

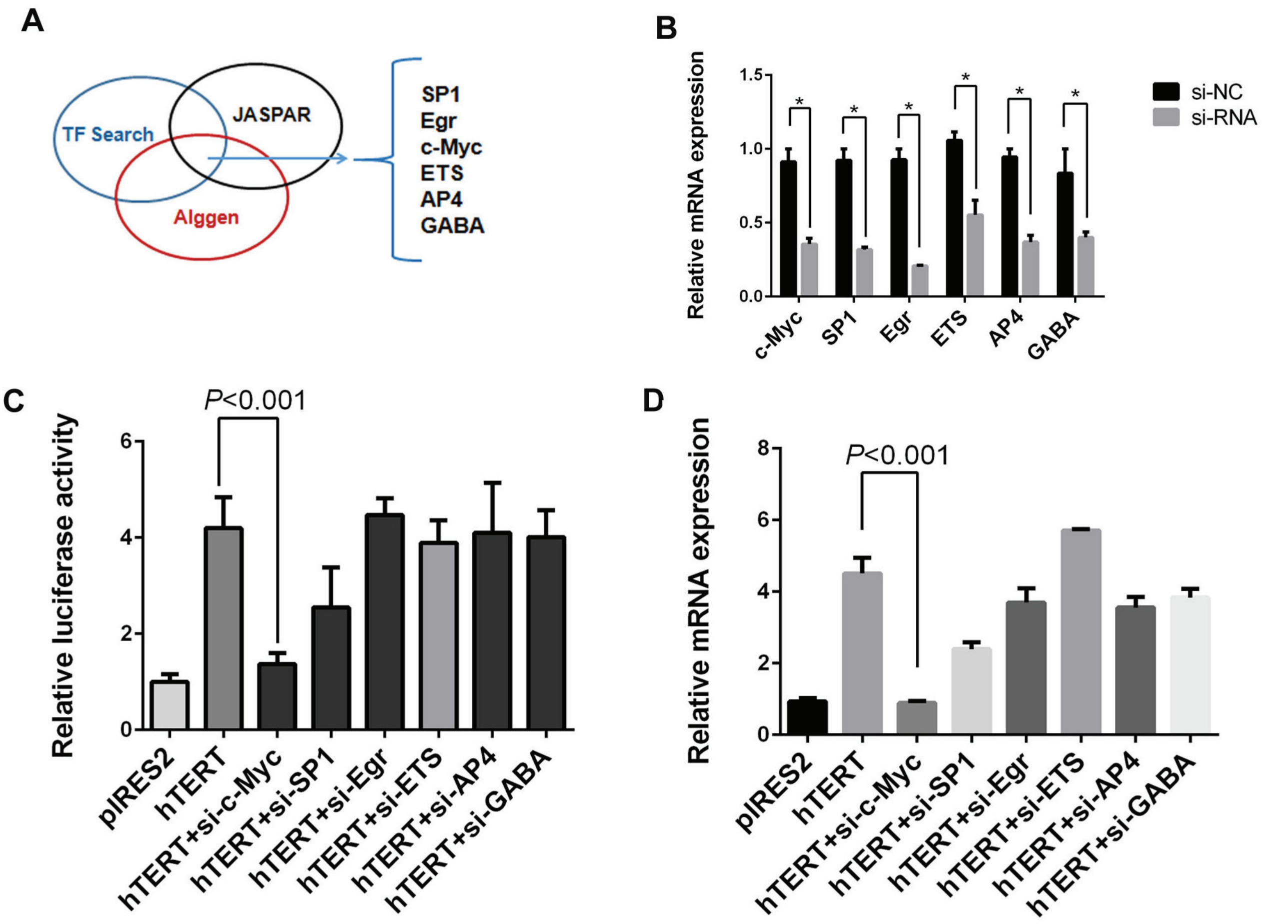


**Supplementary Figure S3: c-Myc as a mediator of hTERT-enhanced transcriptional expression of heparanase. A.** Schematic graph showing the screening of transcription factors using bioinformatics. **B.** The interference efficiency of siRNA was evaluated by qPCR. SGC7901 cells were transfected with siRNAs as indicated. Next, qPCR analysis was performed using specific primers ($^*P < 0.01$). **C.** and **D.** Knockdown of c-Myc was able to impair hTERT-enhanced promoter activity and mRNA expression of heparanase. SGC7901 cells were transiently transfected with hTERT plasmid and siRNAs as indicated. The luciferase assay and qPCR were performed to detect the promoter activity and mRNA expression of heparanase ($P < 0.001$).

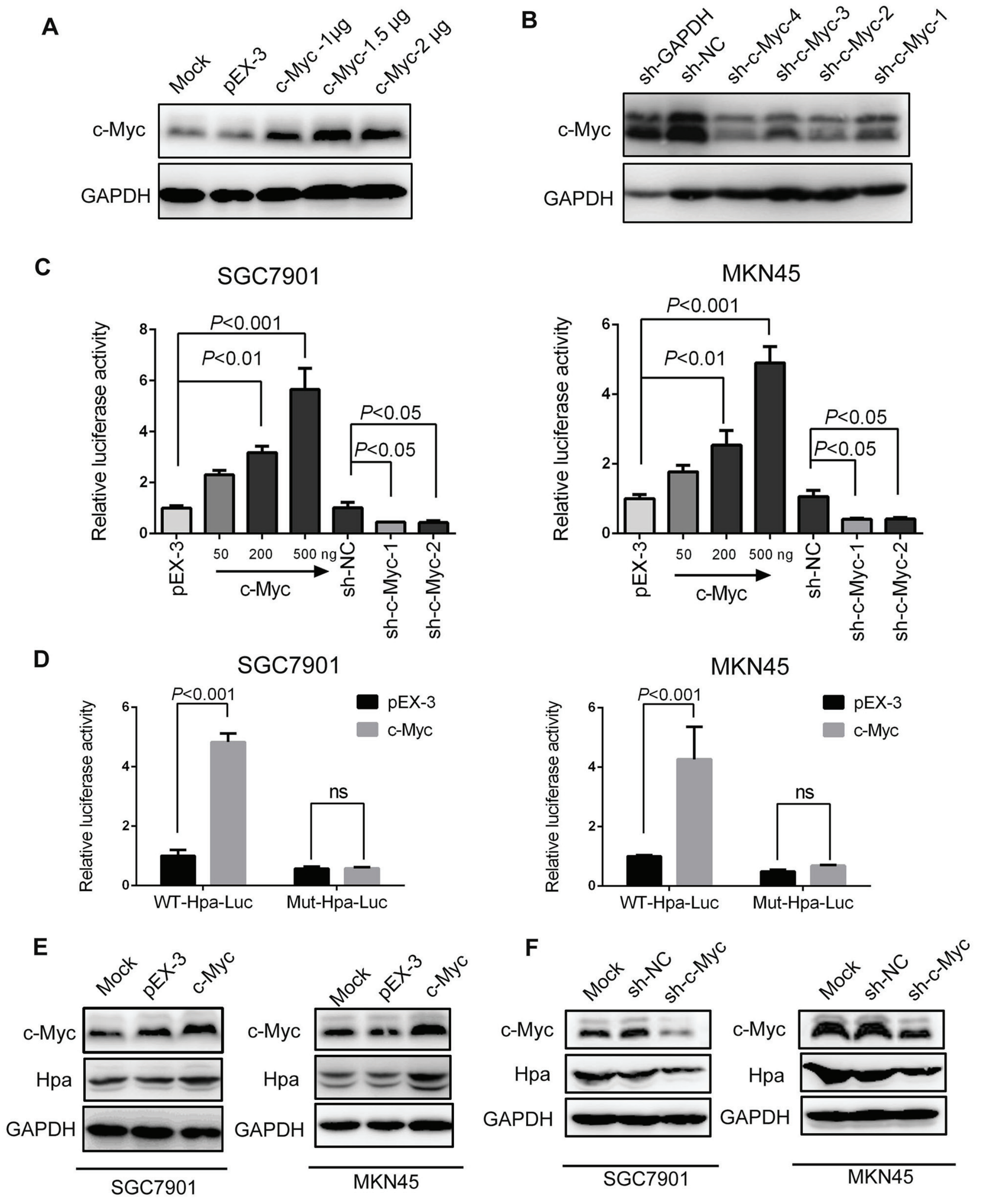


**Supplementary Figure S4: c-Myc regulated the promoter activity and expression of heparanase. A.** and **B.** The protein expression levels of c-Myc were evaluated by western blot analysis. Cells were transfected with c-Myc and sh-c-Myc vectors, respectively. **C.** The relative luciferase activities of the heparanase promoter were tested using the Dual Luciferase assay in SGC7901 and MKN45 cells that were transiently transfected with the pEX-c-Myc (50, 200, 500 ng) and sh-c-Myc-1 and sh-c-Myc-2 vectors, respectively. **D.** The luciferase activities of the heparanase promoter with wild type (or the mutant binding site of c-Myc) were determined using luciferase reporter assays in SGC7901 and MKN45 cells that were transiently transfected with pEX-3 or c-Myc plasmids. All of the experiments were performed at least three times (ns denotes no significance). **E.** and **F.** c-Myc regulated the protein expression of heparanase. SGC7901 and MKN45 cells were transiently transfected with pEX-c-Myc or sh-c-Myc vectors as indicated, respectively. The protein expression levels of c-Myc and heparanase were assessed by western blot analysis.

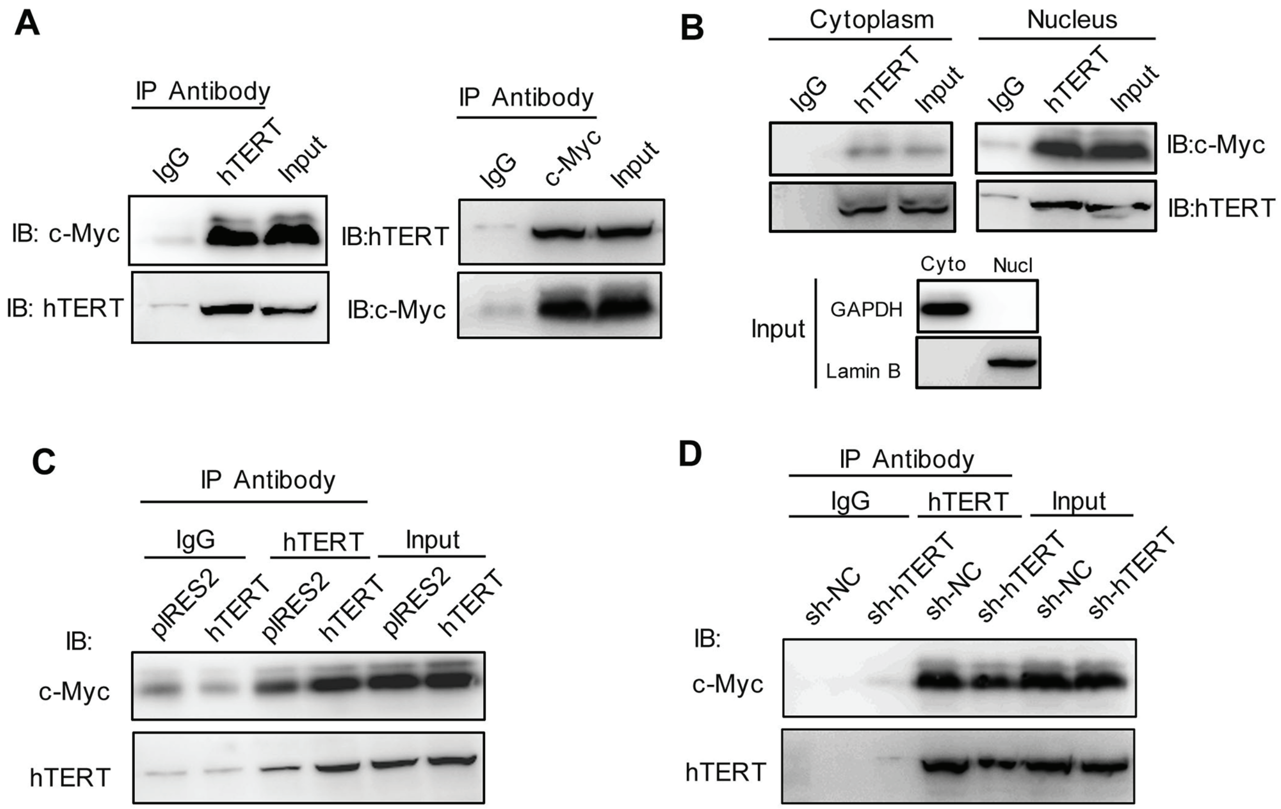


**Supplementary Figure S5: hTERT interacted with c-Myc and regulated the formation of the complex. A.** The total cell lysates of MKN45 cells were prepared for immunoprecipitation (IP) with antibodies against hTERT or c-Myc and then analyzed by immunoblot using antibodies against c-Myc or hTERT, respectively. Rabbit IgG served as a negative control. An aliquot of cell lysate was used as an Input control. **B.** Cytoplasm and nuclear lysates of MKN45 cells were prepared for IP with anti-hTERT or anti-c-Myc and then evaluated by IB. Rabbit IgG served as a negative control. An aliquot of cell lysate was used as an Input control. GAPDH and Lamin B were used as a loading control to examine the purity of the fractions. **C.** and **D.** MKN45 cells were transfected with pIRES2, pIRES2-hTERT plasmids, or sh-RNA, or sh-hTERT vectors, respectively. Next, Co-IP was performed and evaluated with IB using the indicated antibodies. Rabbit IgG was used as a negative control. An aliquot of cell lysate was used as an Input control.

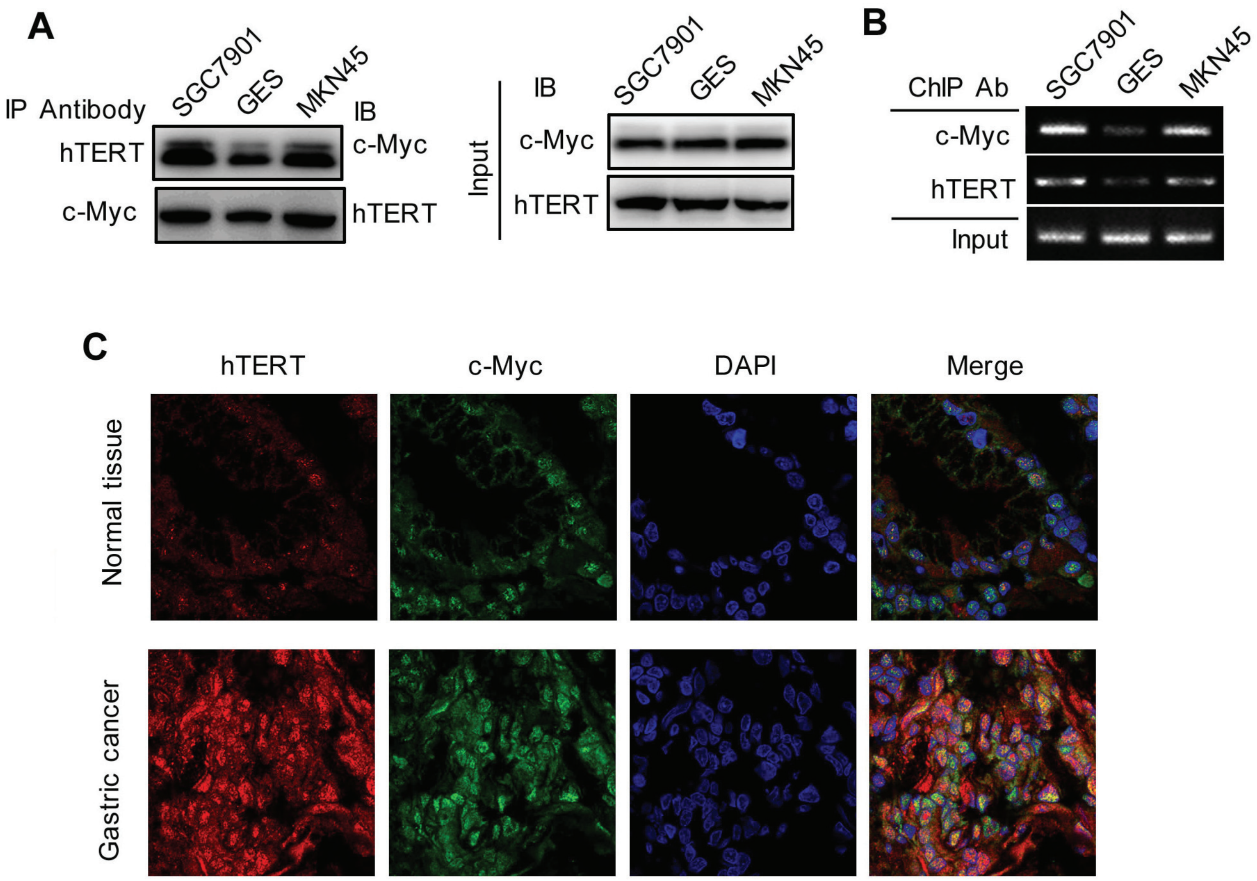


**Supplementary Figure S6: The interaction of hTERT with c-Myc in gastric cancer cells and gastric cancer tissues. A.** Immunoprecipitation (IP) with antibodies against hTERT or c-Myc was performed in normal gastric GES cells, SGC7901 and MKN45 cells, and then immunoblotting was performed using antibodies against c-Myc or hTERT, respectively. Rabbit IgG was used as a negative control. An aliquot of the cell lysate served as an Input control. **B.** ChIP assays were conducted using anti-hTERT or anti-c-Myc antibodies in normal gastric GES cells, SGC7901 and MKN45 cells. The PCR products from the heparanase promoter were separated in 2% agarose gels. An equivalent amount of DNA in all of the samples served as an Input control. **C.** Colocalization of hTERT and c-Myc in the corresponding adjacent normal gastric tissues and gastric cancer tissues was tested by dual immunofluorescence using confocal microscopy. The colocalization of hTERT and c-Myc is shown in yellow in the merged images.

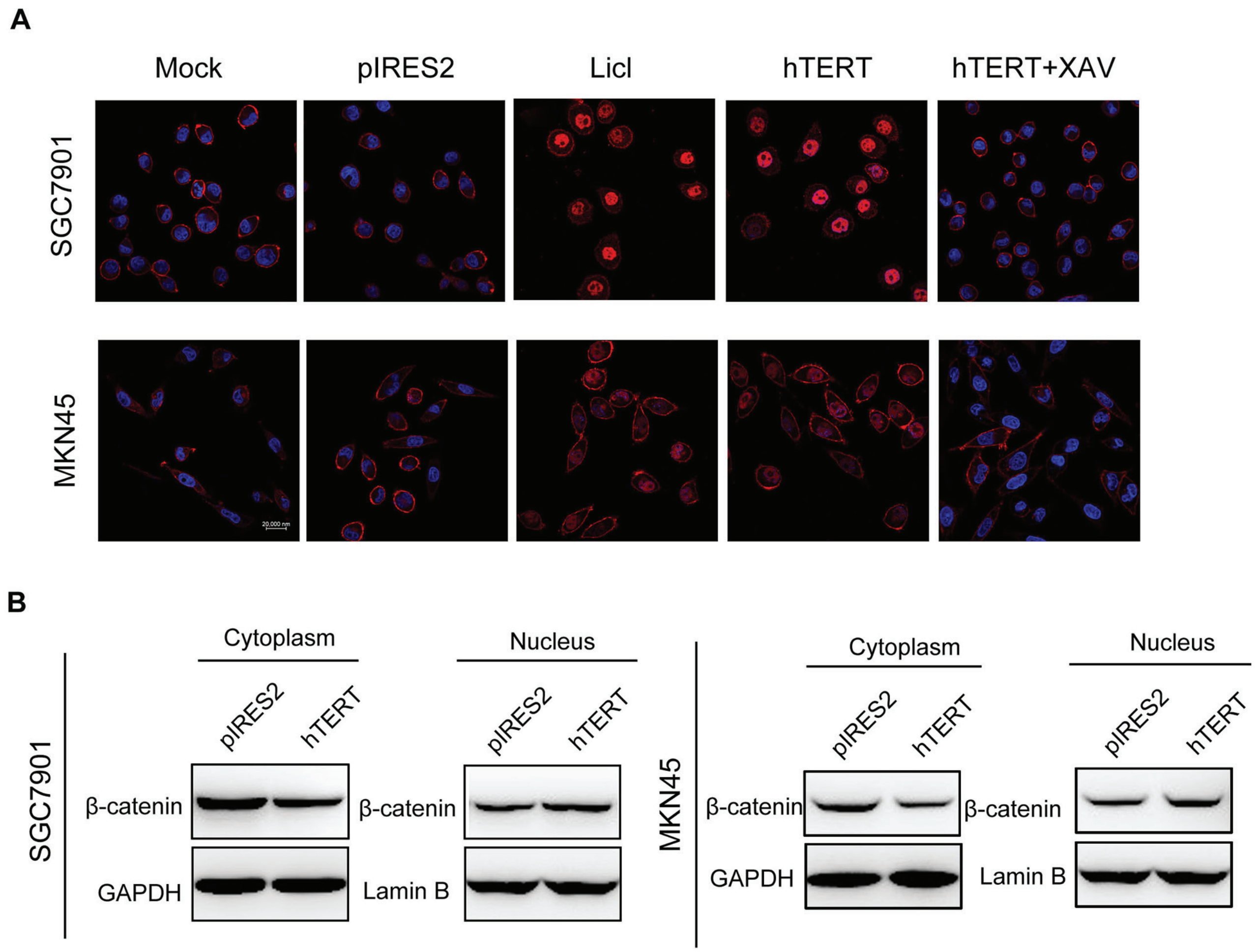


**Supplementary Figure S7: hTERT promoted the nuclear import of β-catenin. A.** The localization of β-catenin in SGC7901 and MKN45 cells transiently transfected with hTERT plasmids and treated with LiCl or XAV939 as indicated was tested by immunofluorescence using confocal microscopy. **B.** Cytoplasm and nuclear lysates of SGC7901 and MKN45 cells were prepared for western blot analysis using anti-hTERT or anti-c-Myc antibodies. GAPDH and Lamin B served as loading controls.

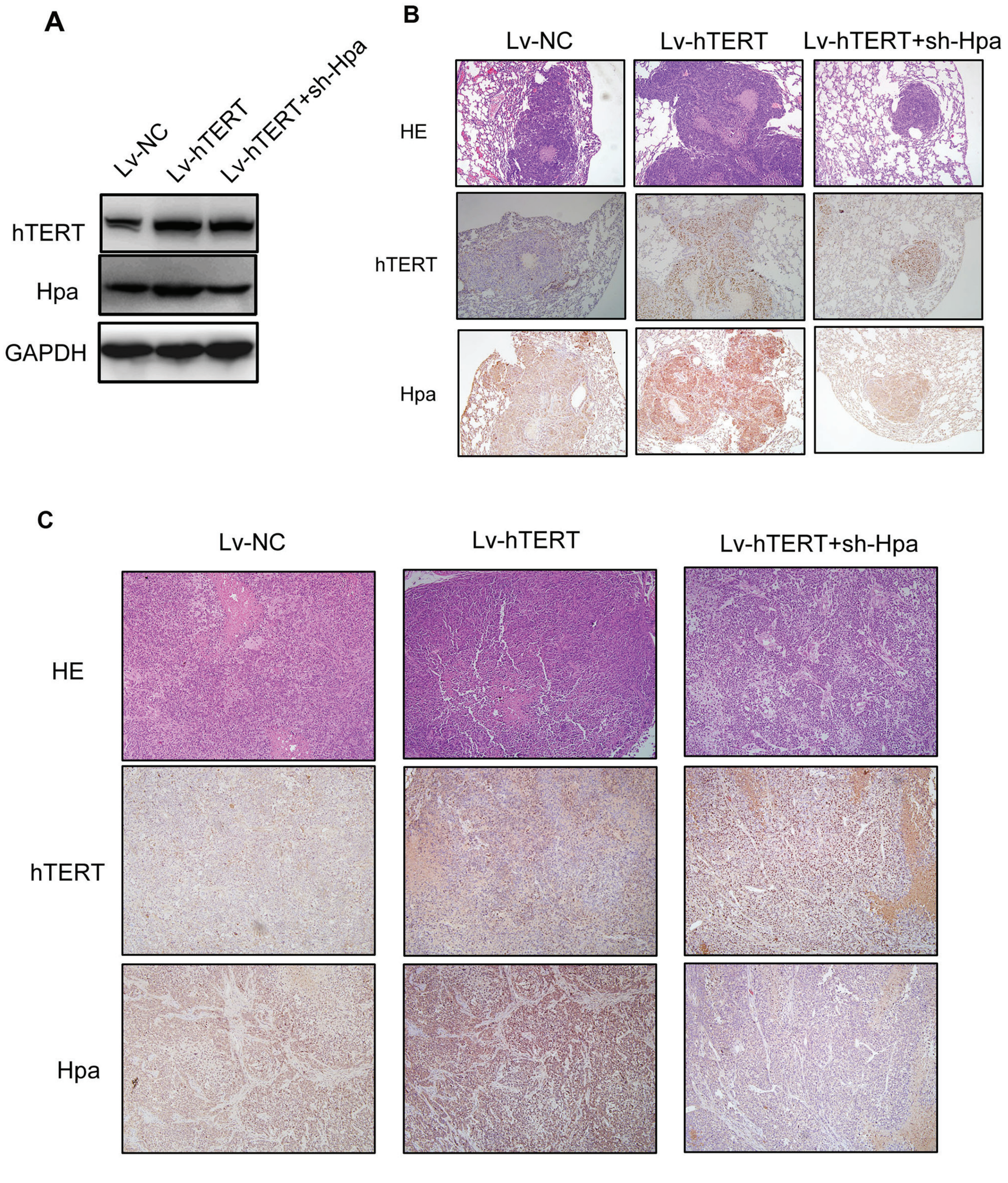


**Supplementary Figure S8: The protein expression level in gastric cancer cells and tissues. A.** Western blot analysis for the protein expression of hTERT and Hpa in tumor nodules from peritoneal dissemination. **B.** SGC7901 cells transfected with the indicated vectors were injected into the tail vein of nude mice. The mice were killed, and the lungs were isolated for hematoxylin and eosin as well as immunohistochemical staining for hTERT and Hpa. **C.** SGC7901 cells transfected with the indicated vectors were injected into nude mice. The tumor nodules from peritoneal dissemination were collected and embedded for hematoxylin and eosin as well as immunohistochemical staining for hTERT and Hpa.

**Supplementary Table S1: Sequences of shRNAs**

| shRNAs | Sequences |
|---|---|
| sh-hTERT-1 | 5′-GCTTCCTCAGGAACACCAAGATTCAAGAGATCTTGGTGTTCCTGAGGAAGC-3′ |
| sh-hTERT-2 | 5′-GGAAGAGTGTCTGGAGCAAGTTTCAAGAGAACTTGCTCCAGACACTCTTCC-3′ |
| sh-c-Myc-1 | 5′-GCCGTATTTCTACTGCGACGATTCAAGAGATCGTCGCAGTAGAAATACGGC-3′ |
| sh-c-Myc-2 | 5′-GCTTGTACCTGCAGGATCTGATTCAAGAGATCAGATCCTGCAGGTACAAGC-3′ |
| sh-c-Myc-3 | 5′-GAGAATGTCAAGAGGCGAACATTCAAGAGATGTTCGCCTCTTGACATTCTC-3′ |
| sh-c-Myc-4 | 5′-GAACAGCTACGGAACTCTTGTTTCAAGAGAACAAGAGTTCCGTAGCTGTTC-3′ |
| sh-Hpa-1 | 5′-GCATCACTACTATTTGAATGGTTCAAGAGACCATTCAAATAGTAGTGATGC-3′ |
| sh-Hpa-2 | 5′-GGCTATCTCTTCTGTTCAAGATTCAAGAGATCTTGAACAGAAGAGATAGCC-3′ |
| sh-Hpa-3 | 5′-GCAAGTGGATAAATACCTTCTTTCAAGAGAAGAAGGTATTTATCCACTTGC-3′ |
| sh-Hpa-4 | 5′-GGACCTCATGGATTACTTTCCTTCAAGAGAGGAAAGTAATCCATGAGGTCC-3′ |
